# Osteosarcoma cell–derived CCL2 facilitates lung metastasis via accumulation of tumor-associated macrophages

**DOI:** 10.1007/s00262-025-04051-x

**Published:** 2025-05-09

**Authors:** Hiroya Kondo, Hiroshi Tazawa, Tomohiro Fujiwara, Aki Yoshida, Miho Kure, Koji Demiya, Nobuhiko Kanaya, Toshiaki Hata, Koji Uotani, Joe Hasei, Toshiyuki Kunisada, Shunsuke Kagawa, Yusuke Yoshioka, Toshifumi Ozaki, Toshiyoshi Fujiwara

**Affiliations:** 1 Departments of Orthopaedic Surgery, Okayama University Graduate School of Medicine, Dentistry and Pharmaceutical Sciences, Okayama, 700-8558 Japan; 2 Departments of Gastroenterological Surgery, Okayama University Graduate School of Medicine, Dentistry and Pharmaceutical Sciences, Okayama, 700-8558 Japan; 3 Center for Innovative Clinical Medicine, Okayama University Hospital, 2-5-1 Shikata-Cho, Kita-Ku, Okayama, 700-8558 Japan; 4 Departments of Sports Medicine, and Okayama University Graduate School of Medicine, Dentistry and Pharmaceutical Sciences, Okayama, 700-8558 Japan; 5 Departments of Medical Materials for Musculoskeletal Reconstruction, Okayama University Graduate School of Medicine, Dentistry and Pharmaceutical Sciences, Okayama, 700-8558 Japan; 6 Department of Molecular and Cellular Medicine, Tokyo Medical University, Tokyo, 160-0023 Japan

**Keywords:** Osteosarcoma, Lung metastasis, Tumor-associated macrophage, CCL2, Extracellular vesicle

## Abstract

Osteosarcoma (OS) is the most common malignant tumor of bone in children and adolescents. Although lung metastasis is a major obstacle to improving the prognosis of OS patients, the underlying mechanism of lung metastasis of OS is poorly understood. Tumor-associated macrophages (TAMs) with M2-like characteristics are reportedly associated with lung metastasis and poor prognosis in OS patients. In this study, we investigated the metastasis-associated tumor microenvironment (TME) in orthotopic OS tumor models with non-metastatic and metastatic OS cells. Non-metastatic and metastatic tumor cells derived from mouse OS (Dunn and LM8) and human OS (HOS and 143B) were used to analyze the TME associated with lung metastasis in orthotopic OS tumor models. OS cell–derived secretion factors were identified by cytokine array and enzyme-linked immunosorbent assay (ELISA). Orthotopic tumor models with metastatic LM8 and 143B cells were analyzed to evaluate the therapeutic potential of a neutralizing antibody in the development of primary and metastatic tumors. Metastatic OS cells developed metastatic tumors with infiltration of M2-like TAMs in the lungs. Cytokine array and ELISA demonstrated that metastatic mouse and human OS cells commonly secreted CCL2, which was partially encapsulated in extracellular vesicles. In vivo experiments demonstrated that while primary tumor growth was unaffected, administration of CCL2-neutralizing antibody led to a significant suppression of lung metastasis and infiltration of M2-like TAMs in the lung tissue. Our results suggest that CCL2 plays a crucial role in promoting the lung metastasis of OS cells via accumulation of M2-like TAMs.

**Supplementary Information:**

The online version contains supplementary material available at 10.1007/s00262-025-04051-x.

## Introduction

Osteosarcoma (OS) is the most common malignant primary tumor of bone in children and adolescents [1, 2]. As the standard treatments for OS, precision surgery, multi-agent chemotherapy, and a combination of these therapies have led to improved long-term survival in more than 60% of patients who initially presented with localized disease [3]. However, limited progress has been made in improving survival outcomes in patients with distant metastasis [4]. More than 80% of patients with metastatic OS have lung metastases [4]. Surgical resection is the only curative treatment for oligometastatic spread to the lungs, in addition to the chemotherapy regimen recommended for those with local OS. However, the prognosis of metastatic OS patients is still poor, and efforts to develop therapeutic strategies to treat lung metastasis have been unsuccessful. Therefore, a better understanding of the precise mechanism leading to lung metastasis is needed.

Tumor-associated macrophages (TAMs) are a major component of tumor-infiltrating immune cells in the tumor microenvironment (TME) [5, 6]. TAMs play a crucial role in the malignant progression of various types of cancer [5, 6], including bone and soft-tissue sarcomas [7, 8]. TAMs secrete growth factors and cytokines that promote tumor angiogenesis, invasion, and metastasis [5, 6]. Macrophages are classified as either proinflammatory M1-like or immunosuppressive M2-like. TAMs display a pro-tumorigenic phenotype associated with an M2-like profile [5, 6]. The TME of sarcomas contains an abundance of infiltrating TAMs [7, 8]. TAMs with the features of an M2-like phenotype promote the proliferation, migration, and invasion of OS cells [7, 8]. Of note, the association between the accumulation of TAMs and prognosis of OS patients remains to be fully elucidated [8]. Moreover, the precise role of TAMs in the development of lung metastasis of OS is poorly understood.

Metastatic organotropism is a crucial factor in the development of metastasis to specific organs in various types of cancer [9]. This phenomenon is a non-random process affected by cancer type, molecular characteristics, the TME, and cross-talk between tumor cells and the TME. Tumor cells secrete many different cytokines and chemokines that modulate the TME, especially development of the pre-metastatic niche (PMN), a metastasis-promoting TME that facilitates the development of metastasis in specific organs [10, 11]. Recent evidence has suggested a potential role for extracellular vesicles (EVs) secreted from tumor cells in formation of the PMN [12, 13]. A recent report showed that tumor cells secrete EVs containing a variety of cytokines and chemokines. The PMN is associated with the infiltration of immune cells, including macrophages [11]. Macrophages play an important role in the TME not only at the primary site but also at sites of metastasis [11]. Therefore, we hypothesized that metastatic OS cells modulate the TME, especially the accumulation of TAMs, leading to development of metastatic tumors via the secretion of metastasis-promoting factors.

In the present study, we evaluated the metastasis-promoting role of the TME and underlying mechanism in the development of lung metastasis of OS using orthotopic tumor models with non-metastatic and metastatic OS cells derived from mice (Dunn, LM8) and humans (HOS, 143B). Primary and metastatic OS tumors were analyzed to evaluate the TME, which is commonly associated with lung metastasis in mouse and human orthotopic OS tumor models. Metastatic OS cell–derived secreted factors were identified using a cytokine array and enzyme-linked immunosorbent assay (ELISA). Orthotopic tumor models with metastatic mouse LM8 and human 143B cells were analyzed to evaluate the therapeutic potential of a neutralizing antibody in the development of primary and metastatic OS tumors.

## Materials and methods

### Cell lines

The murine and human OS cell lines LM8 and 143B were obtained from the Riken BioResource Research Center (Tsukuba, Ibaraki, Japan) and American Type Culture Collection (Manassas, VA, USA), respectively. The Dunn and HOS murine and human OS cell lines were kindly provided by Dr. Hironari Tamiya (Osaka International Cancer Institute, Osaka, Japan) and Dr. Satoru Kyo (Shimane University, Izumo, Japan), respectively. All cells were maintained in modified Eagle’s medium (Sigma-Aldrich, St. Louis, MO, USA) supplemented with 10% fetal bovine serum (FBS), 100 U/mL penicillin, and 100 μg/mL streptomycin. The cells were maintained at 37 °C in a humidified atmosphere with 5% CO_2_. Cells were cultured for no longer than 5 months following resuscitation.

### In vivo orthotopic OS tumor models

Animal experimental protocols were approved by the Ethics Review Committee for Animal Experimentation of Okayama University School of Medicine (OKU-2020833). Dunn and LM8 cells (2 × 10^6^ cells) were inoculated orthotopically into the proximal tibia of 6-week-old female C3H/HeJ mice (CLEA Japan, Tokyo, Japan) (n = 4 in each group). HOS and 143B cells (2 × 10^6^ cells) were orthotopically inoculated into the proximal tibia of 6-week-old female athymic nude mice (CLEA Japan) (n = 4 in each group). Tumor volume was monitored once per week. The weight of murine and human OS tumors was analyzed on days 28 and 35, respectively.

To evaluate the role of CCL2 in lung metastasis, LM8 and 143B cells (2 × 10^6^ cells) were inoculated orthotopically into the proximal tibia of 6-week-old female C3H/He and nude mice, respectively (CLEA Japan) (n = 5 in each group). Armenian hamster anti-mouse/human CCL2-neutralizing antibody (505,915; BioLegend, San Diego, CA, USA) (200 μg/mouse) and isotype control IgG HTK888 (400,967; BioLegend) (200 μg/mouse) were injected intraperitoneally into the mice twice per week. Tumor volume was monitored once per week. The weight of murine and human OS tumors was analyzed on days 24 and 28, respectively.

### Flow cytometry analysis

Flow cytometry was carried out on single-cell suspensions of half of the tumor, lung, and spleen tissues. Erythrocytes in tissues were lysed using red blood cell lysis buffer (420,302; BioLegend). To assess macrophage and monocyte populations, the cell suspension was stained with primary antibodies: Brilliant Violet 421–conjugated rat anti-mouse CD45 monoclonal antibody (mAb) (1:100, 103,134; BioLegend), fluorescein isothiocyanate (FITC)-conjugated rat anti-mouse CD11b mAb (1:200, 101,206; BioLegend), phycoerythrin (PE)/cyanine-7–conjugated rat anti-mouse F4/80 mAb (1:100, 123,114; BioLegend), PE-conjugated rat anti-mouse CD206 mAb (1:200, 141,706; BioLegend), and Alexa Fluor 647–conjugated Armenian hamster anti-mouse CD80 mAb (1:200, 104,718; BioLegend) after blocking Fc receptors using rat anti-mouse CD16/32 mAb (1:200, 156,603, BioLegend) (Table S1). Zombie NIR (423,106; BioLegend) was used to detect dead cells.

### Multi-cytokine and chemokine assay

OS cells were seeded at 5 × 10^6^ cells per T75 cell culture flask in 10 mL of FBS-free MEM. After 48 h, the supernatants were collected and filtered through a 0.22-μm filtration unit. Cytokines and chemokines in the supernatants were detected using Proteome Profiler Mouse Cytokine Array Kit Pane A (ARY006; R&D Systems, Minneapolis, MN, USA) and Proteome Profiler Human XL Cytokine Array Kit (ARY022B; R&D Systems) according to the manufacturer’s protocols.

### ELISA

Levels of CCL2 and M-CSF in the supernatants of mouse and human OS cells were assessed using ELISA kits according to the manufacturer’s protocols. The following ELISA kits were used: Mouse CCL2 Quantikine ELISA kit (MJE00; R&D Systems), Human CCL2 Quantikine ELISA kit (DCP00; R&D Systems), Mouse M-CSF Quantikine ELISA kit (MMC00; R&D Systems), and Human M-CSF Quantikine ELISA kit (DMC00B; R&D Systems).

### RNA extraction and RT-qPCR

Total RNA was extracted from sub-confluent cell cultures using an RNeasy kit (74,104; Qiagen, Valencia, CA, USA) according to the manufacturer’s protocol. qPCR was performed on a Quantstudio 1 Real-Time PCR System (Thermo Fisher Scientific Inc., Waltham, MA, USA) using TaqMan™ Universal Master Mix II (4,440,043; Applied Biosystems, Waltham, MA, USA) and appropriate primers. The following probe sets were used: CCL2 (Hs00234140_m1), colony-stimulating factor 1 (CSF1) (Hs00174164_m1), Ccl2 (Mm00441242_m1), Csf1 (Mm00432686_m1), GAPDH (Hs02786624_g1), and Gapdh (Mm99999915_g1) (Applied Biosystems). The mRNA expression levels were normalized according to GAPDH mRNA and calculated using the 2^−ΔΔCT^ method for qPCR analysis.

### Isolation and purification of EVs from OS cells

EVs were isolated and purified from the supernatants of OS cells (1 × 10^7^ cells) that were maintained in FBS-free medium for 48 h. The supernatants were centrifuged at 2,000 × *g* for 10 min to remove cells and debris, followed by centrifugation at 100,000 × *g* for 70 min at 4 °C after filtration. The final supernatant was collected and filtered through a 0.22-μm filtration unit. The pellets were washed with phosphate-buffered saline (PBS) and centrifuged at 100,000 × *g* for 70 min at 4 °C. The particle size distribution of EVs (40 μL) was determined using dynamic light scattering (DLS) on a Zeta sizer nano ZSP system (Malvern Panalytical, Malvern, UK). Protein concentrations were determined using a Pierce™ Bicinchoninic Acid Protein Assay kit (23,225; Thermo Fisher Scientific) according to the manufacturer’s protocol. The morphology and structure of EVs were observed using transmission electron microscopy (H-7560; Hitachi, Japan) at 80 kV.

### Western blot analysis

Proteins (5 μg) extracted from whole-cell lysates or EVs were electrophoresed on 10% SDS–polyacrylamide gels, transferred onto polyvinylidene difluoride membranes (Hybond-P; GE Healthcare, Buckinghamshire, UK), and then blocked with Blocking-One (03953-95; Nacalai Tesque) at room temperature for 30 min. The membranes were incubated overnight at 4 °C with primary antibodies against mouse and human CCL2 (1:500, MA5-17,040; Invitrogen, Rockford, IL, USA), mouse CD9 (1:500, ab82390; Abcam, Cambridge, UK), mouse CD81 (1:500, MA5-32,333; Invitrogen), human CD9 (1:1000, 13,174; Cell Signaling Technology, Danvers, MA, USA), human CD81 (1:5000, ab79559; Abcam), and mouse and human β-actin (1:5000, A5441; Sigma-Aldrich) (Table S2), followed by incubation with secondary antibodies for 1 h at room temperature. Amersham ECL Prime Western Blotting Detection Reagent (RPN2232; GE Healthcare) was used to detect immunoreactive bands on the blots.

### Fluorescence microscopy

Murine and human OS cells were fixed in 4% paraformaldehyde (PFA), permeabilized with 0.2% Tween 20 at 4 °C, and incubated overnight at 4 °C with primary antibodies against mouse CCL2 (1:200, MA5-17,040; Invitrogen), human CCL2 (1:200, HPA019163; Sigma-Aldrich), mouse CD9 (1:500, 14-0091-82; Invitrogen), and human CD81 (1:500, ab79559, Abcam) (Table S3). After blocking with Blocking-One (03953-95; Nacalai Tesque) and incubation with secondary antibodies for 1 h at room temperature, DNA was stained using 4’,6-diamidino-2-phenylindole (1:1000, D1306; Invitrogen). Cells were observed using an LMS 780 laser-scanning confocal microscope (Carl Zeiss, Jena, Germany). Confocal microscopy images were analyzed using Zen Blue software (ZEISS, v1.1.2.0).

### Immuno-electron microscopy of EVs

Armenian hamster anti-CCL2 mAb 2H5 (505,915; Biolegend) and isotype control IgG HTK888 (400,967; Biolegend) were conjugated with 15-nm NHS-activated gold nanoparticles (NPs) using a Gold Nanoparticle Conjugation kit (CGN5K-15–2; Cytodiagnostics Inc, Burlington, ON, Canada) according to the manufacturer’s protocol. EV samples were then placed on formvar-coated grids and incubated for 15 min at room temperature. After blocking, the EVs were stained with conjugated secondary antibodies for 3 h at room temperature, and then the grid was washed, fixed with 2% PFA, and negatively stained using uranyl acetate. Finally, the grids were examined using transmission electron microscopy (H-7560; Hitachi, Japan) at 80 kV.

### In vivo tracking of DiD-labeled EVs

EVs from murine OS cells (Dunn and LM8) were labeled using Vybrant™ DiD Cell-Labeling Solution (V-22887; Invitrogen) according to the manufacturer’s protocol. DiD-labeled EVs were injected into the tail veins of 6-week-old C3H/HeJ mice (100 μg EVs/mouse). At 3 h after injection, tissues were harvested for ex vivo imaging. The fluorescence intensity was quantified using a Xenogen In Vivo Imaging System (IVIS) Lumina (Caliper Life Sciences, Cheshire, UK) and Living Image Software (PerkinElmer, v4.4) to assess the tissue distribution of DiD-labeled EVs. The average radiant efficiency of EV-injected mice was measured using tissues from PBS-injected control mice (PBS through the same DiD labeling procedure for EVs). DiD-positive immune cells in the lung that had taken up labeled EVs were measured using flow cytometry.

To evaluate the role of CCL2 in the biodistribution of EVs, Armenian hamster anti-mouse/human CCL2–neutralizing antibody 2H5 (505,915; Biolegend; 200 μg/mouse) and isotype control IgG HTK888 (400,967; Biolegend; 200 μg/mouse) were injected intraperitoneally into the mice at 24 h before EV injection and the same time as EV injection.

### Public datasets for sarcoma and identification of immune subsets in the TME

We obtained 606 samples from 4 datasets (GSE75885 [14] and GSE71121 [15] combined with GSE71118, GSE71119, and GSE71120) and 253 sarcoma RNA-Seq datasets from The Cancer Genome Atlas (TCGA) database. The infiltration of immune cells in the TME of sarcoma tissues was analyzed to determine the proportions of 22 types of immune cells based on the normalized gene expression data using the CIBERSORT method. We separated all data into two groups for CCL2 expression, but one of TCGA datasets was excluded because of missing data on CCL2.

### OS patients and clinical samples

The study protocol was approved by the institutional review board at Okayama University Hospital (no. 2108-023). Paraffin-embedded tissues of 31 OS patients were obtained from biopsy samples collected at the time of diagnosis before preoperative chemotherapy in Okayama University Hospital between 2000 and 2018. Informed consent was obtained according to the opt-out method.

### Immunohistochemistry

Tissue sections of the 31 OS patients were heated for antigen retrieval in 10 mM sodium citrate buffer (pH 6.0) and then incubated overnight at 4 °C with primary antibodies against human CCL2 (1:200, MA5-17,040; Thermo Fisher Scientific), human CD68 (1:400, 76,437; Cell Signaling Technology, Danvers, MA, USA), human CD163 (1:500, ab182422; Abcam), human CD80 (1:1000, ab134120; Abcam), or isotype-matched control antibodies (Table S4). Immunodetection was performed using Histofine Simple Stain MAX PO (Nichirei, Tokyo, Japan) and a DAB Substrate kit (Nichirei) according to the manufacturer’s instructions. Sections were counterstained with hematoxylin to create contrast. The percentage of DAB-positive area (ten 200 × images/sample) was determined using the IHC profiler plugin [16] for ImageJ.

### Statistical analyses

GraphPad Prism version 8.0 (GraphPad Software, San Diego, CA, USA) was used for statistical analyses. Data are presented as mean ± SD for results obtained from at least three independent experiments. Mean differences were compared using Student’s *t*-test and one-way ANOVA with Tukey’s post-test for multiple comparisons. Correlations between CCL2 expression and macrophage markers were analyzed using Pearson’s correlation coefficients. Statistical significance was defined as a *P* value of less than 0.05.

## Results

### Comparison of lung metastasis and macrophage accumulation in orthotopic tumor models with non-metastatic and metastatic OS cells

**Fig. 1:**
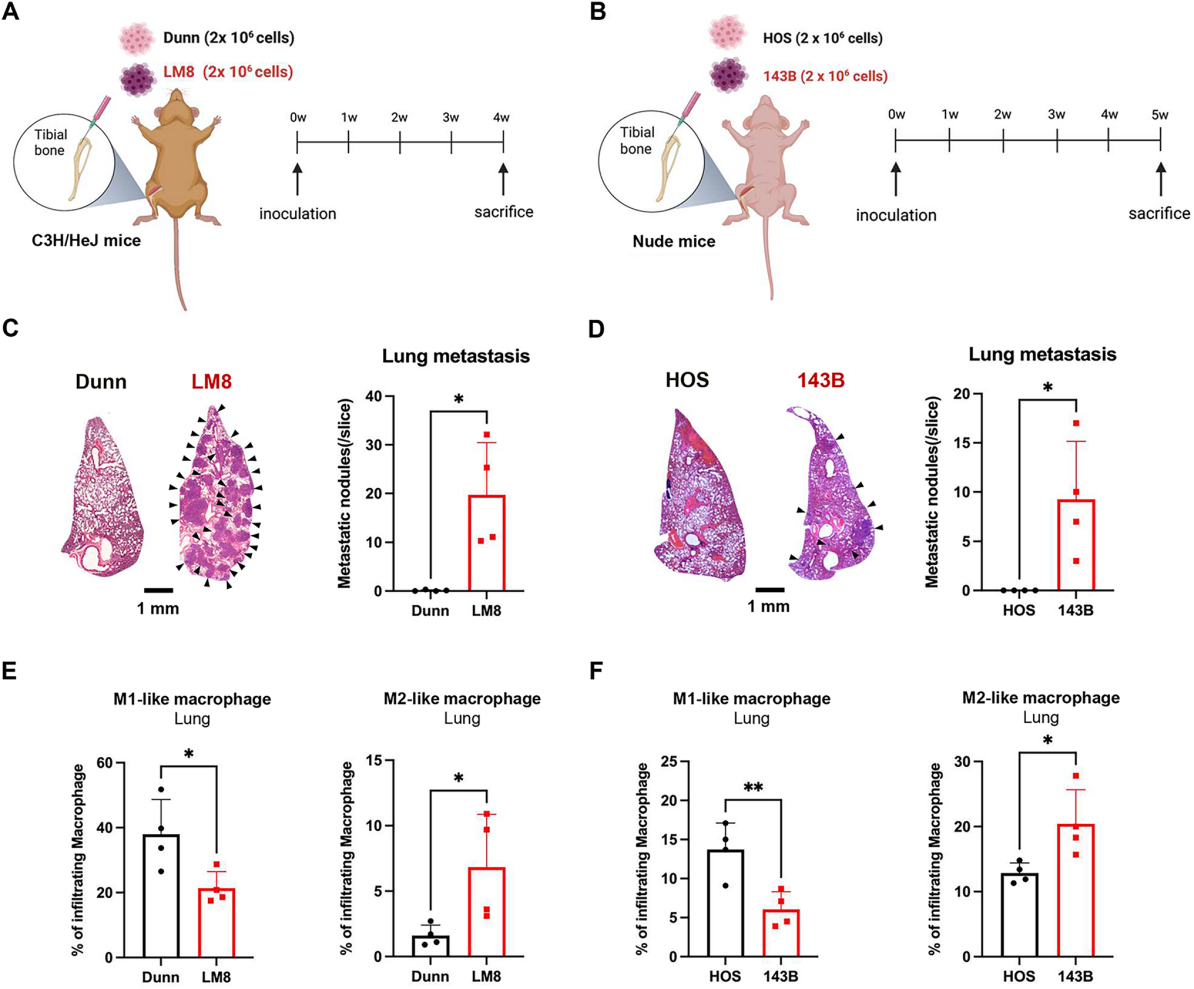
Differential induction of lung metastasis and M2-like macrophage accumulation in orthotopic tumor model mice with non-metastatic and metastatic OS cells. A, B Mouse (**A**) and human (**B**) OS cells with different metastatic potentials (2 × 10^6^ cells) were inoculated into the tibia of C3H/HeJ mice and athymic nude mice, respectively. Lung metastases and the immune microenvironment in mice that received orthotopic inoculation with mouse and human OS cells were assessed on days 28 and 35 after tumor inoculation, respectively. **C**, **D** Left panel: representative photographs of lung tissues stained with hematoxylin/eosin at low magnification. Right panel: number of metastatic nodules in lung tissues is shown as the mean ± SD (n = 4 in each group; *, *P* < 0.05). **E**, **F** Comparison of the polarization of macrophages in lung tissues. The percentages of M1-like and M2-like macrophages are shown as mean ± SD (n = 4 in each group; *, *P* < 0.05; **, *P* < 0.01). Statistical significance was determined using Student’s *t*-test. Figures were generated using BioRender

To evaluate the TME in association with lung metastasis of OS cells, we used orthotopic tumor models with non-metastatic and metastatic OS cells from mice (Dunn and LM8) [17] and humans (HOS and 143B) [18]. LM8 cells are metastatic OS cells derived from non-metastatic Dunn cells, whereas 143B cells are metastatic OS cells derived from non-metastatic HOS cells. Mouse and human OS cells with different metastatic potentials were orthotopically inoculated into the tibia of immune-competent C3H/HeJ mice and immune-deficient nude mice, respectively (Fig. 1A and B). The growth of primary tumors was analyzed until 4 or 5 weeks after tumor cell inoculation (Fig. 1A and B). Dunn and LM8 cells developed primary tumors at similar growth rates, and there was no significant difference in the tumor weight between Dunn and LM8 tumors (Fig. S1A and B). By contrast, 143B cells developed primary tumors, although HOS cells did not (Fig. S1C and D). Metastatic LM8 and 143B cells developed lung metastasis, whereas non-metastatic Dunn and HOS cells did not (Fig. 1C and D).

To evaluate the TME in association with lung metastasis, primary tumor, lung, and spleen tissues of mice with or without lung metastasis were next evaluated by FACS analysis. M1-like and M2-like macrophages were identified by the expression of CD80 and CD206, respectively, within the population of CD45 + CD11b + F4/80 + cells (Fig. S2). The proportion of M1-like macrophages was significantly higher in LM8 tumors compared with Dunn tumors, whereas 143B tumors showed a higher proportion of M1-like macrophages than M2-like macrophages (Fig. S3A and B). Lung tissues with metastatic LM8 and 143B tumors showed significantly increased accumulation of M2-like macrophages and a lower proportion of M1-like macrophages compared with tissues without metastatic tumors (Fig. 1E and F). An increased proportion of M2-like macrophages was observed in the spleen tissues of mice with metastatic 143B tumors, whereas spleen tissues of mice with metastatic LM8 tumors showed a significantly decreased proportion of M2-like macrophages (Fig. S3C and D). These results suggest that mouse and human OS cells with metastatic potential commonly promote the accumulation of M2-like macrophages in lung tissues.

### CCL2 is commonly secreted by mouse and human OS cells with metastatic potential

**Fig. 2:**
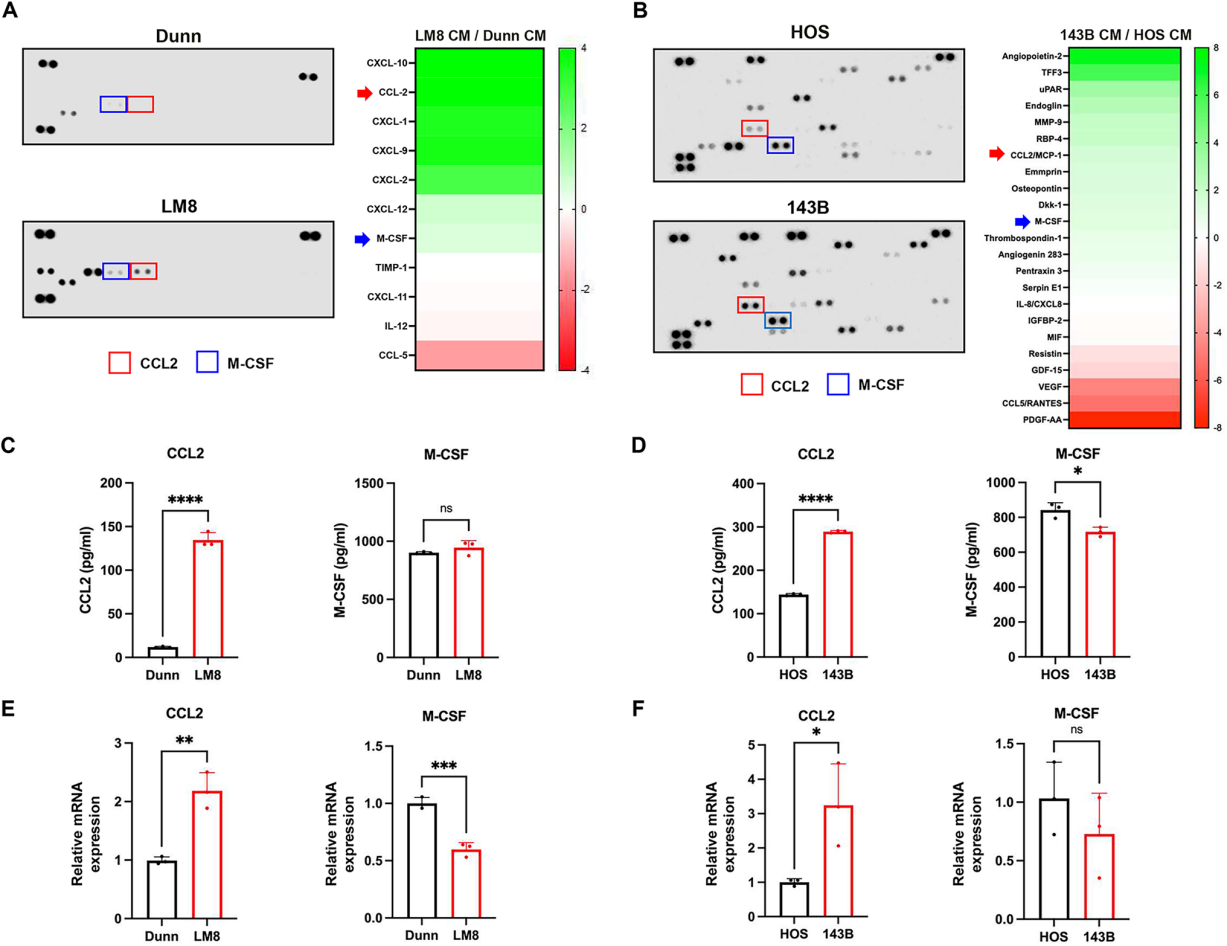
Identification of CCL2 commonly secreted by mouse and human OS cells with metastatic ability. **A**, **B** Conditioned medium (CM) of mouse (**A**) and human (**B**) OS cells was subjected to cytokine/chemokine array analysis. **C**, **D** The amounts of CCL2 and M-CSF in the CM were analyzed by ELISA. The mRNA expression of CCL2 and M-CSF in OS cells was analyzed by RT-PCR. Data are expressed as mean ± SD (n = 3 in each group; *, *P* < 0.05; **, *P* < 0.01; ***, *P* < 0.001; ****, *P* < 0.0001; ns, not significant). Statistical significance was determined using Student’s *t*-test

To identify the metastatic OS cell–derived secreted factors that promote the accumulation of M2-like macrophages in lung tissues, we analyzed conditioned medium (CM) of metastatic and non-metastatic OS cells using a comprehensive cytokine and chemokine array. A variety of cytokines and chemokines were differentially secreted by metastatic LM8 and 143B cells compared with non-metastatic Dunn and HOS cells (Fig. 2A and B). Among the cytokines and chemokines secreted by metastatic OS cells, CCL2 and M-CSF were generally secreted at higher levels by metastatic LM8 and 143B cells compared with non-metastatic Dunn and HOS cells (Fig. 2A and B). ELISA results demonstrated that metastatic LM8 and 143B cells secreted a significantly higher amount of CCL2 protein compared with non-metastatic Dunn and HOS cells (Fig. 2C and D). RT-qPCR analysis showed significantly increased expression of CCL2 mRNA in metastatic LM8 and 143B cells compared with non-metastatic Dunn and HOS cells (Fig. 2E and F). By contrast, there were no significant differences in M-CSF secretion and M-CSF mRNA expression between metastatic and non-metastatic OS cells (Fig. 2C–F). These results suggest that metastatic OS cells produce and secrete higher amounts of CCL2 compared with non-metastatic OS cells.

### CCL2 is abundant in EVs secreted by metastatic OS cells

**Fig. 3:**
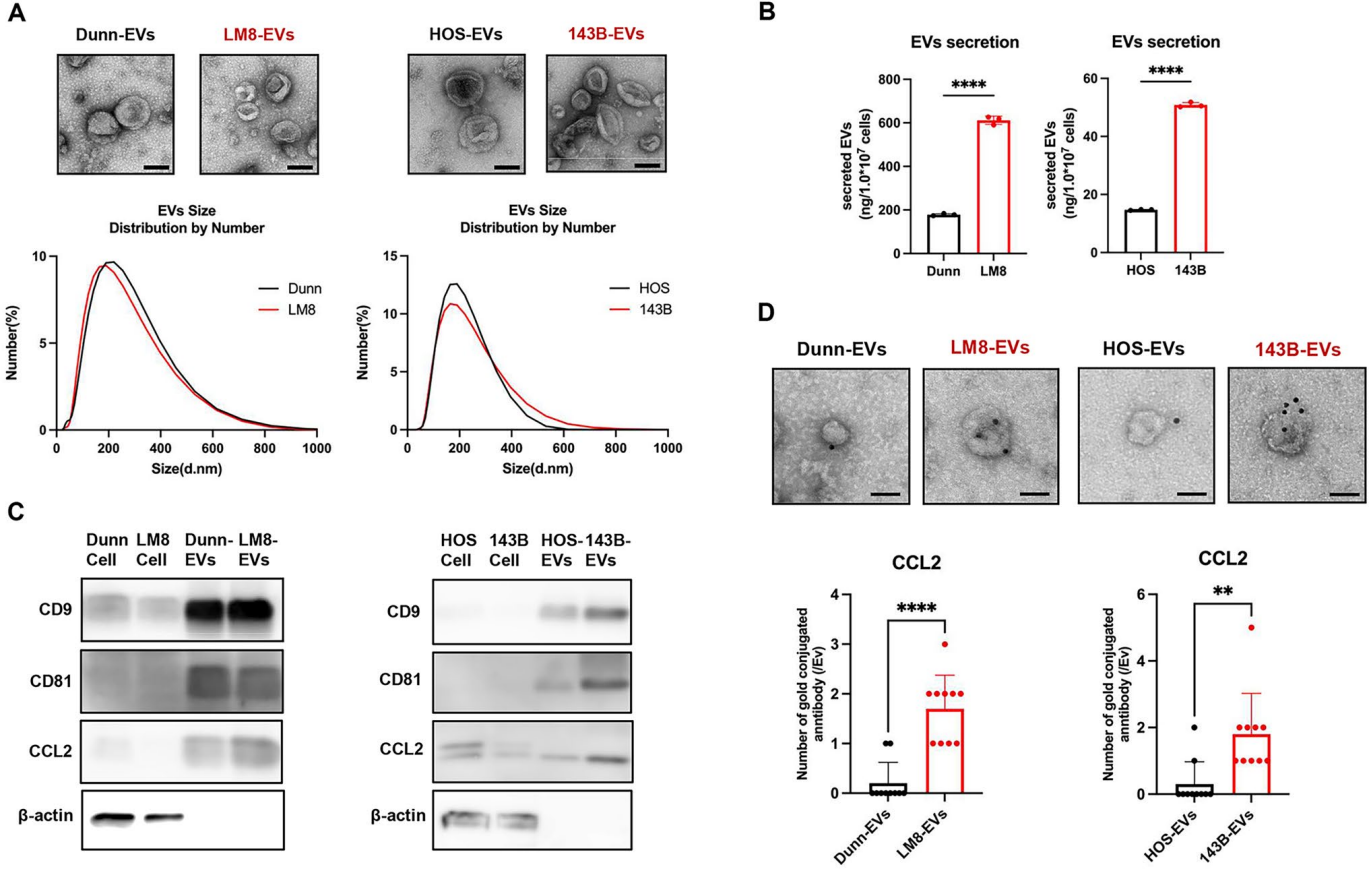
Evaluation of CCL2 expression on EVs secreted by murine and human OS cells with different metastatic potentials. **A** Upper panel: representative images of EVs isolated from murine and human OS cells analyzed by transmission electron microscopy. Lower panel: particle size distribution of EVs measured by dynamic light scattering. **B** Secretion of EVs from murine and human OS cells (1 × 10^7^ cells) was quantified based on the amount of protein (n = 3 in each group; ****, *P* < 0.0001). Statistical significance was determined using Student’s *t*-test. **C** Cell lysates (Cell) and EV lysates (EVs) obtained from mouse and human OS cells were subjected to Western blotting of EV surface markers (CD9, CD81) and CCL2. **D** Upper panel: representative photographs of EVs labeled with anti-CCL2 antibody–conjugated gold particles. Lower panel: number of gold particles in EVs. Data are expressed as mean ± SD (n = 10 in each group; **, *P* < 0.01; ****, *P* < 0.0001). Scale bars, 100 nm

Recent reports have shown that metastatic breast cancer cells secrete CCL2 protein encapsulated in EVs [19]. Therefore, to investigate whether metastatic OS cells secrete CCL2 protein encapsulated in EVs, we isolated the EVs from CM of metastatic and non-metastatic OS cells. The EVs of metastatic and non-metastatic OS cells were similar in terms of morphology and size (Fig. 3A). Metastatic LM8 and 143B cells secreted a significantly higher number of EVs than non-metastatic Dunn and HOS cells (Fig. 3B). Western blot analysis demonstrated that the EVs of OS cells contained high levels of EV marker proteins (CD9, CD81) and that CCL2 protein levels were higher in the EVs of OS cells than in whole-cell lysates of OS cells (Fig. 3C). The localization of CCL2 with isolated EVs was further analyzed using a gold particle–conjugated anti-CCL2 antibody. The number of gold particles conjugated with the anti-CCL2 antibody was significantly higher on EVs of metastatic OS cells compared with EVs of non-metastatic OS cells (Fig. 3D). Consistent with the results of Western blot analyses, fluorescence immunocytochemistry showed co-localization of CCL2 protein with EV markers (CD9 and CD81) in the cytoplasm of metastatic OS cells (Fig. S4). These results suggest that metastatic OS cells partially secrete CCL2 protein encapsulated in EVs.

### EVs secreted by metastatic OS cells preferentially accumulate in the macrophages of lung tissues

**Fig. 4:**
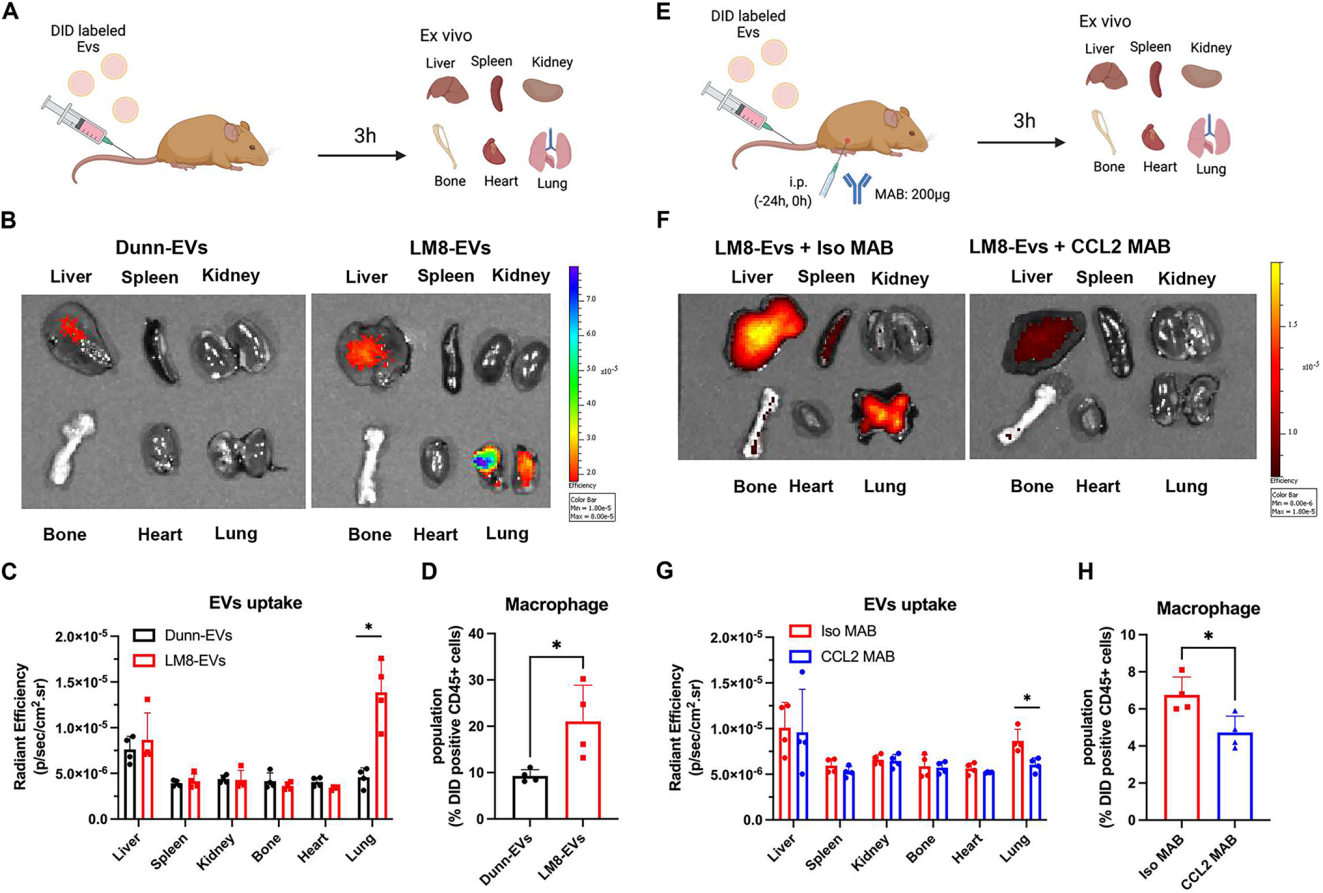
Biodistribution of DiD-labeled EVs secreted by murine OS cells with different metastatic abilities. **A** DiD-labeled EVs of mouse Dunn and LM8 cells (100 mg/mouse) were intravenously injected into the tail vein of mice. The biodistribution of fluorescently labeled EVs was assessed 3 h after injection using an In Vivo Image System. **B** Representative ex vivo images of fluorescence in various organs (liver, spleen, kidney, bone, heart, lung). **C** Quantification of DiD fluorescence in various organs. **D** Percentage of macrophages among DiD + CD45 + cells in lung tissues. **E** CCL2-neutralizing antibody (CCL2 MAB) and isotype control IgG (Iso MAB) (200 ng/mouse) were administered 24 h before and just before injection of EVs. Biodistribution of EVs was assessed 3 h after EV injection. **F** Representative ex vivo images of fluorescence in various organs (liver, spleen, kidney, bone, heart, lung). **G** Quantification of DiD fluorescence in various organs. **H** Percentage of macrophages among DiD + CD45 + cells in the lung tissues. Data are expressed as mean ± SD (n = 4 in each group; *, *P* < 0.05). Statistical significance was determined using Student’s *t-*test. Figures were generated using BioRender

To next evaluate the biodistribution of EVs secreted from metastatic OS cells, EVs isolated from metastatic LM8 and non-metastatic Dunn cells were labeled with the fluorescent dye DiD and intravenously injected into C3H/HeJ mice. After 3 h, tissues of several organs (lung, spleen, kidney, bone, heart) were analyzed by IVIS and flow cytometry (Fig. 4A). Lung tissues contained significantly higher amounts of fluorescently labeled EVs derived from metastatic LM8 cells than EVs derived from non-metastatic Dunn cells (Fig. 4B and C). Additionally, metastatic LM8 cell–derived EVs accumulated in greater numbers in CD45 + F4/80 + macrophages of lung tissues than did Dunn-derived EVs (Fig. 4D). To further evaluate whether CCL2 protein plays a critical role in the accumulation of metastatic OS cell–derived EVs in lung tissues, fluorescently labeled EVs derived from metastatic LM8 cells were intravenously injected into the tail vein of mice that had received an intraperitoneal injection of CCL2-neutralizing antibody or isotype control IgG (Fig. 4E). Administration of CCL2-neutralizing antibody significantly suppressed the accumulation of metastatic LM8 cell–derived EVs in the lung tissues and macrophages compared with isotype control IgG (Fig. 4F-H). These results suggest that the EVs of metastatic OS cells accumulate in the macrophages of lung tissues partially in a CCL2-dependent manner.

### Anti-metastatic effect of CCL2-neutralizing antibody in orthotopic OS tumor models with lung metastasis

**Fig. 5:**
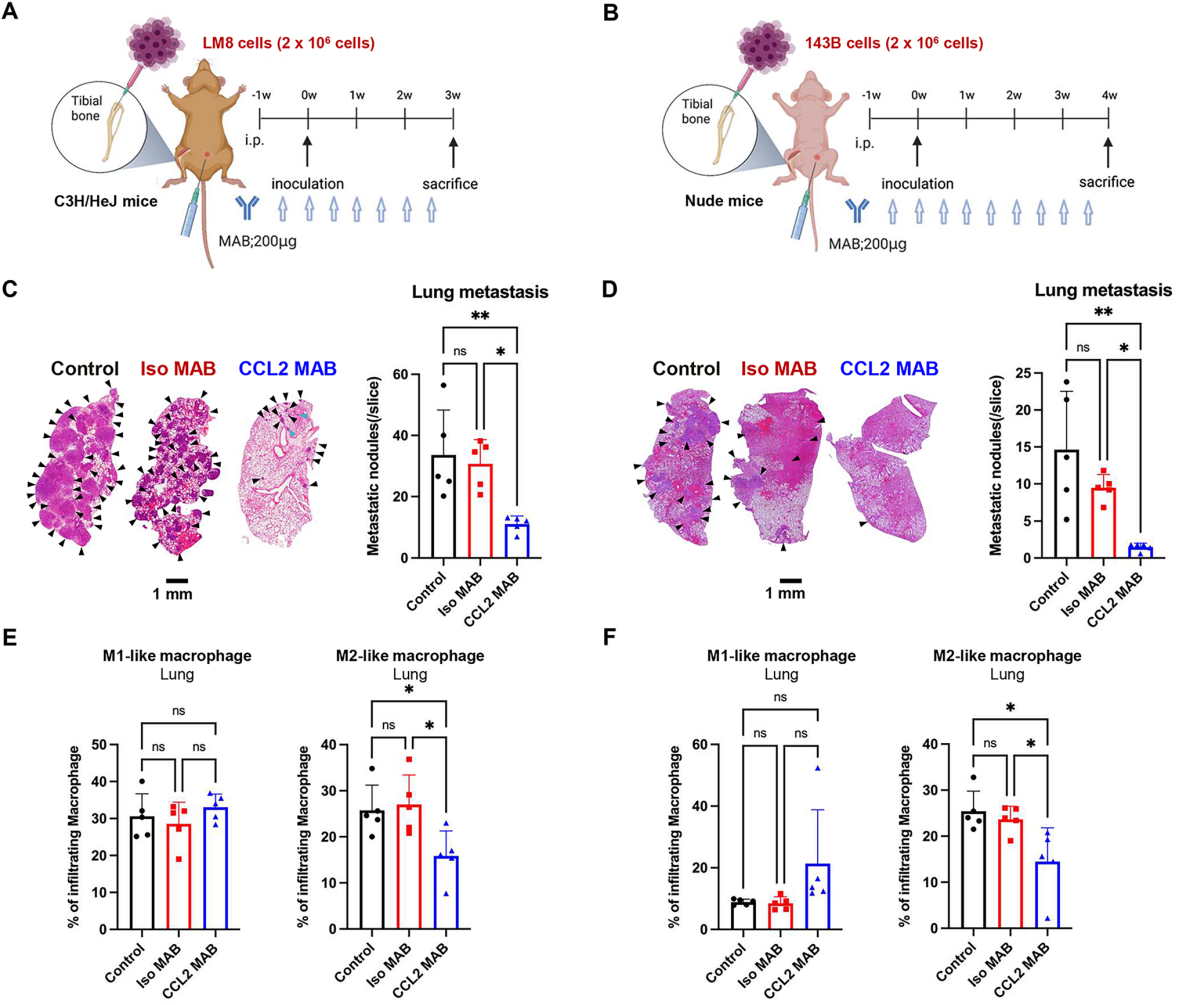
CCL2-neutralizing antibody prevents lung metastasis via suppression of macrophage polarization in the lung. **A**, **B** Mouse LM8 cells (**A**) and human 143B cells (**B**) with metastatic potential (2 × 10^6^ cells) were inoculated into the tibia of C3H/Hej mice and athymic nude mice, respectively. CCL2-neutralizing antibody (CCL2 MAB) and control isotype IgG (Iso MAB) (200 μg/mouse) were administered intraperitoneally twice per week, starting 3 days before inoculation. Lung metastases and the immune microenvironment in mice that received orthotopic inoculation with LM8 and 143B cells were assessed on days 21 and 28 after tumor inoculation, respectively. **C**, **D** Left panel: representative photographs of lung tissues stained with hematoxylin/eosin at low magnification. Right panel: number of metastatic nodules in the lung tissues is shown as mean ± SD (n = 5 in each group; *, *P* < 0.05; **, *P* < 0.01; ns, not significant). **E**, **F** Comparison of the polarization of macrophages in lung tissues. The percentages of M1-like and M2-like macrophages are shown as mean ± SD (n = 5 in each group; *, *P* < 0.05; ns, not significant). Statistical significance was determined using one-way ANOVA with Tukey’s post-test. Figures were generated using BioRender

To evaluate the role of CCL2 in the lung metastasis of OS cells, orthotopic tumor model mice were injected intraperitoneally with CCL2-neutralizing antibody or isotype control IgG along with metastatic LM8 and 143B cells (Fig. 5A and B). Administration of CCL2-neutralizing antibody did not suppress the growth of primary tumors, and there were no significant differences in the weight of primary tumors between mice injected with the CCL2-neutralizing antibody versus the isotype control IgG (Fig. S5). By contrast, administration of CCL2-neutralizing antibody significantly suppressed lung metastasis compared with the isotype control IgG or control groups (Fig. 5C and D). The proportion of immune cells in each tumor was analyzed by flow cytometry. Administration of CCL2-neutralizing antibody significantly suppressed the accumulation of M2-like macrophages in the lung tissues compared with the isotype control IgG group or non-treated control group (Fig. 5E and F). However, there were no significant differences in the accumulation of M2-like macrophages in the primary tumors and spleen (Fig. S6). These results suggest that CCL2 plays a crucial role in the lung metastasis associated with accumulation of M2-like macrophages.

### Clinical significance of CCL2, M2-like macrophages, and lung metastasis in sarcoma tissues

**Fig. 6:**
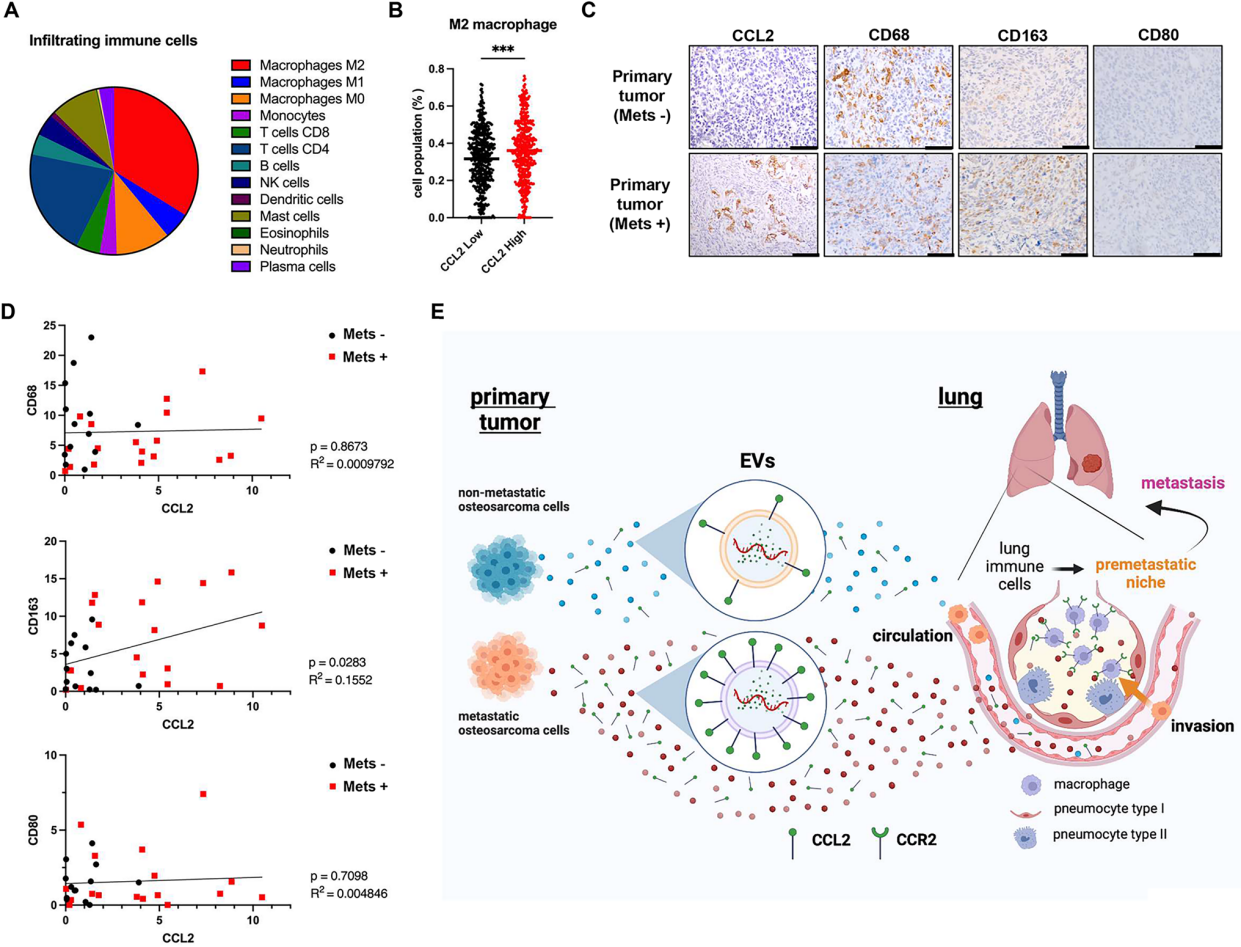
Clinical relevance of the relationship between M2-like macrophages, CCL2, and lung metastasis in sarcomas. **A** Pie chart demonstrating that subsets of 13 immune cell types infiltrated in 858 sarcoma samples. **B** Dot plots showing the correlation between CCL2 expression and M2-like macrophages in sarcoma tissues. Statistical significance was determined using Student’s *t-*test. **C** Immunohistochemistry analysis of CCL2, CD68 (macrophages), CD163 (M2-like macrophages), and CD80 (M1-like macrophages) in primary OS tumors with or without lung metastasis. **D** Correlation between CCL2 expression and expression of CD68 (macrophages), CD163 (M2-like macrophages), and CD80 (M1-like macrophages). Statistical significance was determined using Pearson’s correlation coefficients. **E** Outline describing lung metastasis of OS cells via the accumulation of CCL2 and CCL2 + EVs secreted by metastatic OS cells. Figures were generated using BioRender

Finally, to investigate whether M2-like macrophages are associated with CCL2 expression in sarcoma patients, we evaluated the infiltration of 22 immune cell subsets in sarcoma tissues using CIBERSORT analysis of 868 clinical samples obtained from 5 publicly available RNA-Seq datasets. More than half of the tumor-infiltrating immune cells in sarcoma tissues were myeloid cells, in which M2-like macrophages were the most abundant subset (Fig. 6A). We further evaluated the correlation between CCL2 expression level and accumulation of M2-like macrophages. The CCL2-high group showed significantly greater accumulation of M2-like macrophages compared with the CCL2-low group (Fig. 6B). These results suggest a possible relationship between CCL2 expression and M2-like macrophages in primary sarcoma tissues.

To investigate the relationship between CCL2, M2-like macrophages, and lung metastasis in OS patients, we next analyzed 31 biopsy samples, including primary OS tissues without lung metastasis (n = 13) and primary OS tissues with lung metastasis (n = 18) (Table S5). The expression levels of CCL2, CD68 (pan-macrophages), CD163 (M2-like macrophages), and CD80 (M1-like macrophages) were analyzed using immunohistochemistry. A significant correlation was observed between CCL2 expression and CD163 + expression (M2-like macrophages) (Fig. 6C and D). However, there were no significant correlations between CCL2 expression and CD68 + expression (macrophages) or CD80 + expression (M1-like macrophages) (Fig. 6C and D). These results suggest that CCL2 plays a crucial role in inducing the accumulation of M2-like macrophages in primary OS tumors with metastatic potential.

## Discussion

Overcoming lung metastasis is a major obstacle to improving the clinical outcome of OS patients. However, details regarding the mechanism of lung metastasis in OS remain poorly understood. In this study, we demonstrated that mouse and human OS cells with lung metastatic potential commonly induced the accumulation of M2-like macrophages in the lung tissues in an orthotopic OS tumor model by secreting CCL2 protein, which was partially encapsulated in EVs and preferentially accumulated in macrophages in lung tissues. Intraperitoneal administration of a CCL2-neutralizing antibody significantly suppressed EV accumulation in lung tissues and lung metastasis in orthotopic OS tumor model mice. Moreover, a significant relationship between CCL2 expression and M2-like macrophages was observed in primary and metastatic lung tumors of OS patients. Thus, OS cell–derived CCL2 represents a potent therapeutic target for preventing the lung metastasis of OS.

Mouse LM8 and human 143B cells commonly developed metastatic tumors characterized by the accumulation of M2-like macrophages in the lung tissues of mice that received an orthotopic injection of OS cells (Fig. 1). M2-like macrophages reportedly play a crucial role in the lung metastasis of OS cells [7, 8]. In a mouse LM8 tumor model, Shiraishi et al. demonstrated that lung metastasis of LM8 cells was attenuated in mice with genetic depletion of CD163, an M2-like macrophage marker [20]. Kimura et al. showed that dihydrocoumarins suppress the polarization of M2-like macrophages and lung metastasis in a subcutaneous LM8 tumor model [21]. We also recently reported that PLX3997, a potent inhibitor of the CSF1 receptor, suppresses lung metastasis in orthotopic LM8 tumor models by suppressing the polarization of M2-like macrophages [22]. By contrast, in a human 143B tumor model, Luu et al. demonstrated that orthotopic injection of human 143B cells strongly induces lung metastasis, consistent with our results [23]. Although the precise mechanism of lung metastasis induced by the accumulation of M2-like macrophages in orthotopic LM8 and 143B tumor models remains unclear, M2-like macrophages may play a crucial role in the lung metastasis of LM8 and 143B cells.

Conversely, the proportion of M2-like macrophages was lower in the spleen of metastatic LM8 tumor-bearing mice compared with non-metastatic Dunn tumor–bearing mice (Fig. S3). These findings suggest the modulation of macrophage polarization differs between lung and spleen tissues. In a study of murine breast cancer models with lung metastasis, Qian et al. demonstrated that inflammatory monocytes in the blood are recruited to the lung, but not the spleen, and differentiate into pro-tumoral metastasis-associated macrophages [24]. Serbina et al. reported that circulating inflammatory monocytes are derived from the bone marrow (BM) during bacterial infection [25]. Regarding the polarization of macrophages in the BM and spleen, Zhao et al. showed that BM-derived macrophages polarized primarily toward the M2 phenotype, whereas splenic macrophages (SPMs) exhibit a strong capacity to polarize toward the M1 phenotype [26]. Wang et al. demonstrated that under resting conditions, BM-derived macrophages produce high levels of immunosuppressive cytokines, whereas SPMs maintain high production of pro-inflammatory cytokines [27]. Thus, the polarization of macrophages may be differentially regulated between lung and spleen tissues.

Although we compared only two pairs of OS cell types with non-metastatic versus metastatic potentials, CCL2 was identified as a factor commonly secreted by metastatic LM8 and 143B cells (Fig. 2), and treatment with a CCL2-neutralizing antibody suppressed lung metastasis in orthotopic LM8 and 143B tumor model mice (Fig. 5), suggesting CCL2 plays a crucial role in promoting lung metastasis of OS cells. Consistent with our results, recent accumulating evidence has suggested that CCL2 plays a crucial role in the lung metastasis of breast cancer [28]. Regarding the underlying mechanism of cancer cell–mediated CCL2 secretion, Li et al. demonstrated that genetic inactivation of retinoblastoma increases the secretion of CCL2 by sarcoma and breast cancer cells by activating the JNK signaling pathway [29]. Mu et al. showed that BRD4 activates the expression of CCL2 in human soft-tissue sarcoma cells by activating the NF-kB signaling pathway [30]. Moreover, CCL2 overexpression was shown to be associated with poor prognosis of lung cancer [31], esophageal cancer [32], hepatocellular carcinoma [33], glioblastoma multiforme [34], and diffuse large B-cell lymphoma [35]. Therefore, metastatic tumor cells in several types of cancer may promote tumor progression by activating CCL2-related signaling pathways and subsequent CCL2 secretion.

Cytokine array analyses of the present study identified M-CSF as a metastasis-associated factor differentially secreted between non-metastatic and metastatic OS cells, although there was no significant difference in ELISA results (Fig. 2). As we recently demonstrated that the M-CSF receptor inhibitor pexidartinib suppresses lung metastasis in orthotopic LM8 tumor models [22], M-CSF is expected to be involved in the lung metastasis of OS cells. Moreover, our cytokine array analyses revealed that metastatic OS cells secrete high levels of various CXCR2 ligands, including CXCL1, CXCL2, and CXCL8, than non-metastatic OS cells (Fig. 2), suggesting that the CXCL1/2/8-CXCR2 axis plays a role in the lung metastasis of OS cells. Lee et al. reported that inhibition of CXCL1 in OS cells suppresses lung metastasis in orthotopic human MG63 tumor models [36]. Deng et al. showed that metastatic OS cell–derived EVs induce CXCL2 secretion by lung macrophages to recruit the immunosuppressive myeloid-derived suppressor cells for lung premetastatic niche formation [37]. Gross et al. demonstrated that pharmacological inhibitors of CXCL8 and IL-6 suppress lung metastasis in 143B tumor models [38]. In addition to CCL2, M-CSF and CXCR2 ligands are expected to be involved in the lung metastasis of OS cells.

In this study, CCL2 was partially encapsulated in EVs secreted by mouse LM8 and human HOS cells with metastatic potential (Fig. 3). Mouse and human OS cell–derived CCL2 + EVs preferentially accumulated in CCL2 receptor CCR2 + macrophages in lung tissues (Fig. 4). Several cytokines and adhesion molecules have been shown to be involved in the biodistribution of EVs [39]. CCL2 has been identified as a critical factor on the surface of EVs derived from metastatic breast cancer cells [19]. CCL2 + EVs derived from metastatic breast cancer cells are more likely to be taken up by CCR2 + macrophages in lung tissues, which is consistent with our findings. Several reports have shown that EV uptake leads to the development of a PMN that favors metastasis to the lung [10, 40–42]. Araki et al. demonstrated that genetic inhibition of EV secretion by metastatic LM8 cells prevented lung metastasis in an orthotopic tumor model [43]. Mazumdar et al. showed that metastatic 143B cells induce the accumulation of cancer-associated fibroblasts and inflammatory myeloid cells in lung tissues by secreting EVs [44, 45]. Although whether OS cell–derived CCL2 is primarily present on the surface or within EVs or expressed in an EV-independent manner remains unclear, CCL2 + EVs may partially induce the formation of a PMN in lung tissues by promoting the accumulation of M2-like macrophages.

The underlying mechanism of the CCL2 + EV-mediated polarization of M2-like macrophages remains unclear. The CCL2-CCR2 axis has been shown to be an important factor in the polarization of M2-like macrophages. Sierra-Filardi et al. demonstrated that activation of the CCL2-CCR2 axis induces the differentiation of anti-inflammatory M2-like macrophages, whereas suppression of the CCL2-CCR2 axis leads to enhanced expression of M1-polarization genes and cytokines [46]. In particular, the JAK/STAT signaling pathway has been shown to play a crucial role in the polarization of M2-like macrophages [47]. By contrast, recent accumulating evidence has shown that EVs derived from metastatic murine OS cells (K7M2, K7M3, LM8) and human OS cells (MG63) induce the polarization of M2-like macrophages [48–50]. Li et al. showed that EVs obtained from clinical OS tumors promote the polarization of M2-like macrophages via activation of the STAT6 signaling pathway [51]. Therefore, further studies are warranted to evaluate whether CCL2 + EVs induce the polarization of M2-like macrophages by inducing CCL2- and EV-mediated signaling pathways.

It is worth noting that intraperitoneal administration of a CCL2-neutralizing antibody significantly suppressed lung metastasis in our study but not primary tumor growth in orthotopic tumor model mice (Fig. 5). Consistent with our results, other reports have shown that treatment with a CCL2-neutralizing antibody or genetic knockout of CCL2 suppresses lung metastasis, but not primary tumor growth, in in vivo models involving mouse and human breast cancer cells [28]. These findings suggest the potential limitations of monotherapy with CCL2-neutralizing antibodies as an adjuvant therapy. Targeting the CCL2-CCR2 axis has been shown to modulate the TME and responsiveness to immune checkpoint inhibitors [52]. Tu et al. demonstrated that combination therapy involving CCR2 antagonism and anti-programmed cell death protein 1 (PD-1) antibody suppresses primary tumor growth and lung metastasis in several murine tumor models by activating antitumor immune responses [53]. Therefore, further studies are warranted to evaluate the therapeutic potential of combination therapy with inhibitors of the CCL2-CCR2 axis and immunotherapy in terms of suppressing the growth of primary and metastatic OS tumors.

Recent clinical data suggest that there is a strong relationship between CCL2 expression and poor prognosis in various types of cancer, including breast cancer [54, 55]. A correlation between CCL2 expression and lung metastasis has also been shown in breast cancer patients [28]. In this study, we demonstrated that there is a significant relationship between CCL2 expression and M2-like macrophages in primary tumors and lung metastasis (Fig. 6). Several reports have shown that monitoring CCL2 levels in the blood would be useful as a biomarker for predicting prognosis in cancer patients [54].

Current research progress on NP-based approaches has shown that NPs have great potential for use in the diagnosis and treatment of malignant bone tumors [56]. With regard to diagnosis, Wang et al. demonstrated that the metastasis-specific DNA aptamer LP-16 binds to metastatic 143B cells but not non-metastatic U-2OS cells [57], which could facilitate the development of efficient approaches to target metastatic OS cells. EVs are predominant intercellular crosstalk mediators within the TME [58]. As EVs represent a novel diagnostic biomarker and therapeutic target for evaluating and modulating metastatic potential in OS patients [59], NP-based strategies for evaluating the abundance of CCL2 + EVs in blood samples could represent a promising approach for preventing lung metastasis in OS patients. With regard to treatment, NPs overcome the limitation of conventional antitumor modalities associated with toxicity risks by exhibiting improved drug retention and permeability at tumor sites [56]. Various types of NPs are currently utilized as drug carriers and immune activators to promote tumor immunotherapy [60–63]. Cao et al. showed that macrophage membrane–coated emtansine liposomes suppress lung metastasis in a murine breast cancer model by enhancing specific targeting to sites of lung metastasis [64]. Yin et al. demonstrated that macrophage membrane–coated NPs exhibiting enhanced PD-1 expression suppress tumor growth in a murine glioblastoma tumor model by activating antitumor immune responses [65]. Therefore, macrophage membrane–coated NP-based strategies appear promising for use in antitumor therapies to treat lung metastasis in OS patients.

In conclusion, we demonstrated that murine and human OS cells with metastatic potential commonly secrete CCL2 partially encapsulated in EVs, which induces the accumulation of M2-like macrophages in lung tissues, thereby contributing to the development of lung metastasis. Taken together, our data suggest that targeting the CCL2-mediated accumulation of M2-like macrophages would be a promising antitumor strategy for preventing the lung metastasis of OS cells.

## Supplementary Information

Below is the link to the electronic supplementary material.

Supplementary file1 (PDF 1196 KB)

Supplementary file2 (XLSX 18 KB)

Supplementary file3 (XLSX 18 KB)

Supplementary file4 (XLSX 18 KB)

Supplementary file5 (XLSX 18 KB)

Supplementary file6 (XLSX 18 KB)

## Data Availability

No datasets were generated or analysed during the current study.
