# Supplementary material for: Osteosarcoma cell–derived CCL2 facilitates lung metastasis via accumulation of tumor-associated macrophages
Source: Cancer Immunol Immunother. 2025 May 9;74(7):193. doi: 10.1007/s00262-025-04051-x (PMC12064505; doi:10.1007/s00262-025-04051-x)
Supplement: Supplementary file 1 — Supplementary file1 (PDF 1196 KB) [file 262_2025_4051_MOESM1_ESM.pdf]

Supplementary Information

Osteosarcoma cell-derived CCL2 facilitates lung metastasis via accumulation of tumor-associated macrophages

Hiroya Kondo[1], Hiroshi Tazawa[2,3], Tomohiro Fujiwara[1,4], Aki Yoshida[1], Miho Kure[1], Koji Demiya[1], Nobuhiko Kanaya[2], Toshiaki Hata[1], Koji Uotani[1], Joe Hasei[1], Toshiyuki Kunisada[1,5], Shunsuke Kagawa[2], Yusuke Yoshioka[6], Toshifumi Ozaki[1],Toshiyoshi Fujiwara[2]

Departments of [1]Orthopaedic Surgery, [2]Gastroenterological Surgery, [4]Sports Medicine, and [5]Medical Materials for Musculoskeletal Reconstruction, Okayama University Graduate School of Medicine, Dentistry and Pharmaceutical Sciences, Okayama 700-8558, Japan; [3]Center for Innovative Clinical Medicine, Okayama University Hospital, Okayama 700-8558, Japan. [7]Department of Molecular and Cellular Medicine, Tokyo Medical University, Tokyo 160-0023, Japan.

Supplementary Figure S1
Tumor growth curves and tumor weights in orthotopic tumor models with non-metastatic and metastatic OS cells.

Supplementary Figure S2
Gating strategy for M1-like and M2-like macrophages in FACS analysis.

Supplementary Figure S3
Proportion of M1-like and M2-like macrophages in primary tumors and spleens of mice bearing non-metastatic and metastatic OS tumors.

Supplementary Figure S4
Immunohistochemistry analysis of CCL2 and EV surface markers (CD8, CD81) using mouse and human OS cells.

Supplementary Figure S5
Gating strategy for macrophages containing fluorescence-labeled EVs in FACS analysis.

Supplementary Figure S6
Tumor growth curves, tumor weights, and body weights in orthotopic tumor models with metastatic OS cells after treatment with CCL2-neutralizing antibody.

Supplementary Figure S7
Proportion of M1 and M2 macrophages in primary tumors and spleens of mice bearing metastatic OS tumors after treatment with CCL2-neutralizing antibody.

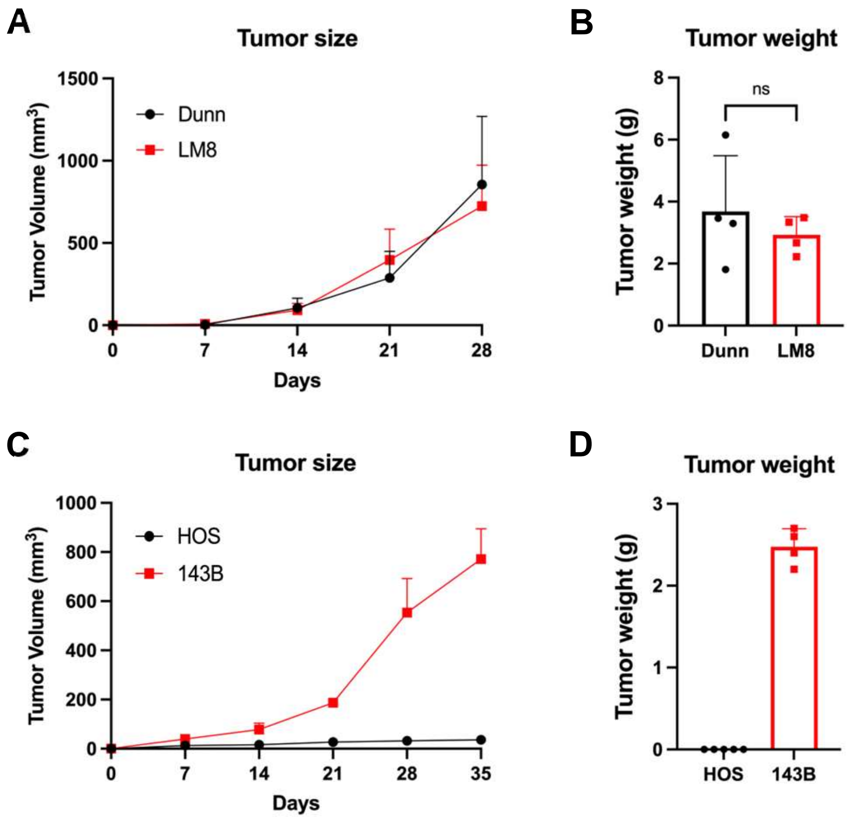

Supplementary Figure S1. Tumor growth curves and tumor weights in orthotopic tumor models with non-metastatic and metastatic OS cells. **A** Murine OS cells with different metastatic abilities (non-metastatic Dunn and metastatic LM8) (2×10^6 cells) were inoculated orthotopically into the tibia of C3H/He mice. Tumor volumes were monitored once a week until 28 days after tumor inoculation. **B** Mean weights of Dunn and LM8 tumors on day 28. **C** Human OS cells with different metastatic abilities (non-metastatic HOS and metastatic 143B) (2×10^6 cells) were inoculated orthotopically into the tibia of nude mice. Tumor volumes were monitored once a week until 35 days after tumor inoculation. **D** Mean weights of HOS and 143B tumors on day 35. Data are expressed as mean $\pm$ SD (n = 4 in each group; ns, not significant).

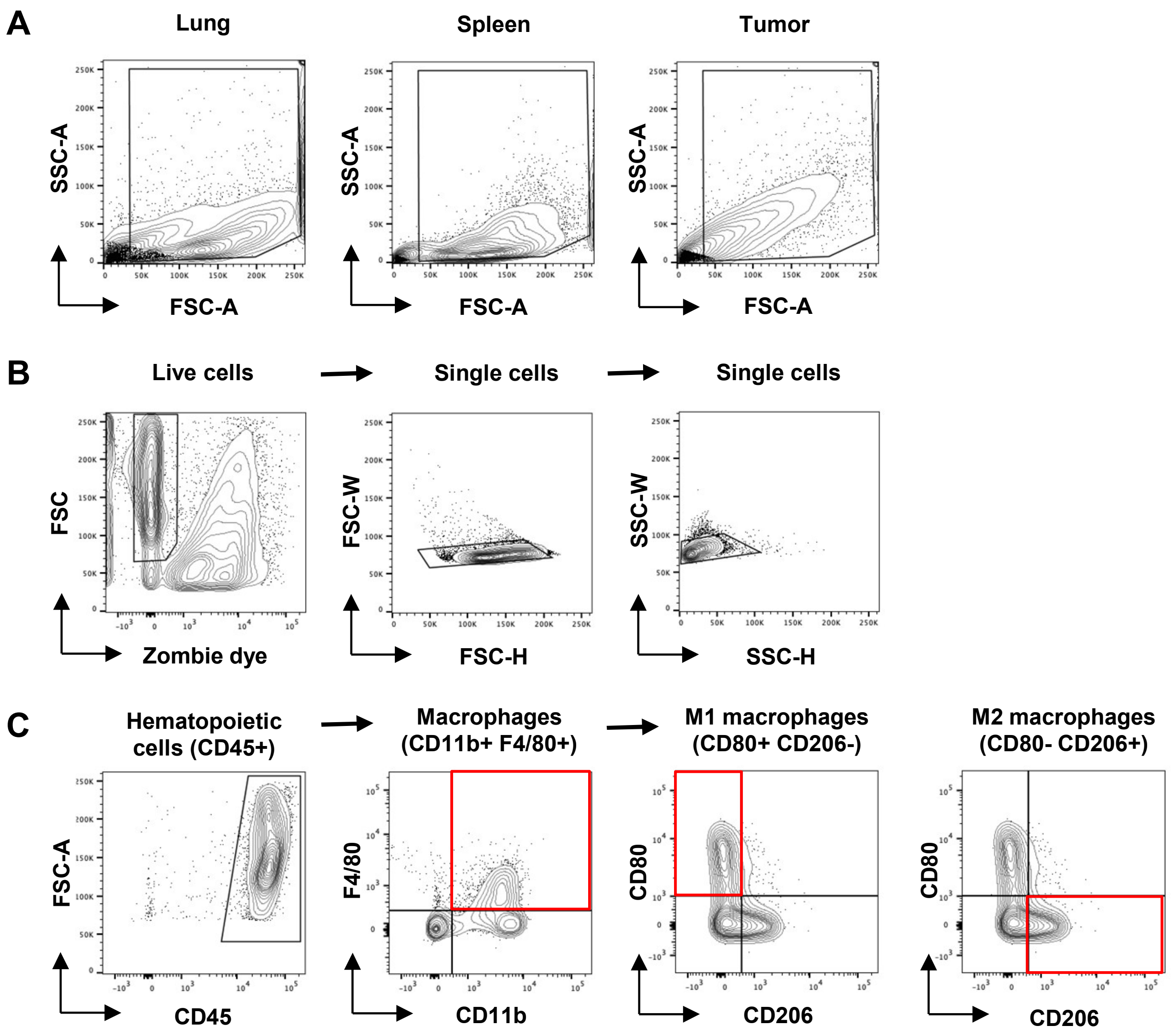

Supplementary Figure S2. Gating strategy for M1-like and M2-like macrophages in FACS analysis. **A** Representative FSC and SSC plots with gating for live cells. **B** Representative FSC, SSC, and zombie dye plots with gating for single live cells. **C** Representative FSC, CD45, CD11b, F4/80, CD206, and CD80 plots with gating for hematopoietic cells, macrophages, M1-like macrophages, and M2-like macrophages.

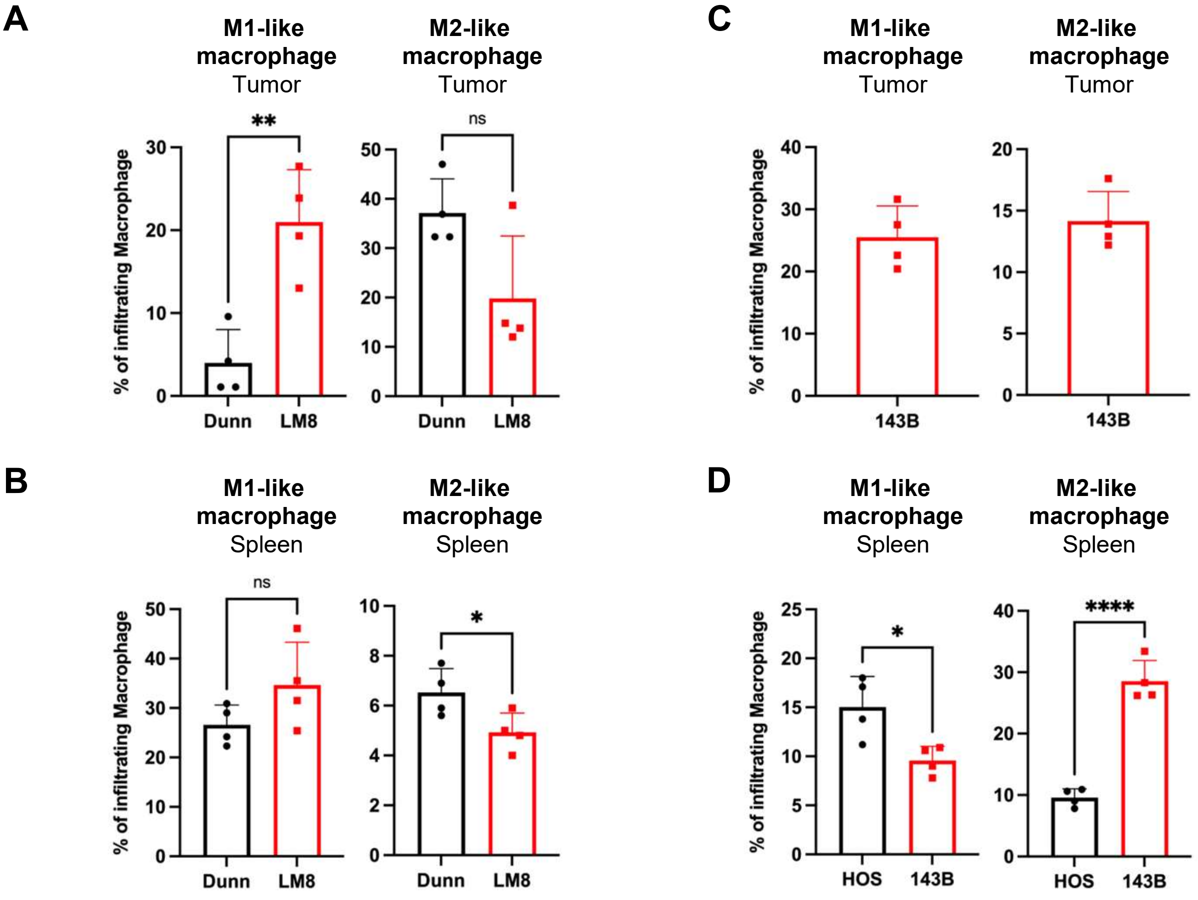

Supplementary Figure S3. Proportions of M1-like and M2-like macrophages in primary tumors and spleens of mice bearing non-metastatic and metastatic OS tumors. A, **B** The proportions of M1 and M2 macrophages in primary tumors (A) and spleens (B) of mice orthotopically inoculated with Dunn and LM8 cells. **C**,**D**, The proportions of M1 and M2 macrophages in primary tumors (C) and spleens (D) of mice orthotopically inoculated with HOS and 143B cells. Data are expressed as mean ± SD (n = 4 in each group; *, $P < 0.05$; **, $P < 0.01$; **** $P < 0.0001$; ns, not significant).

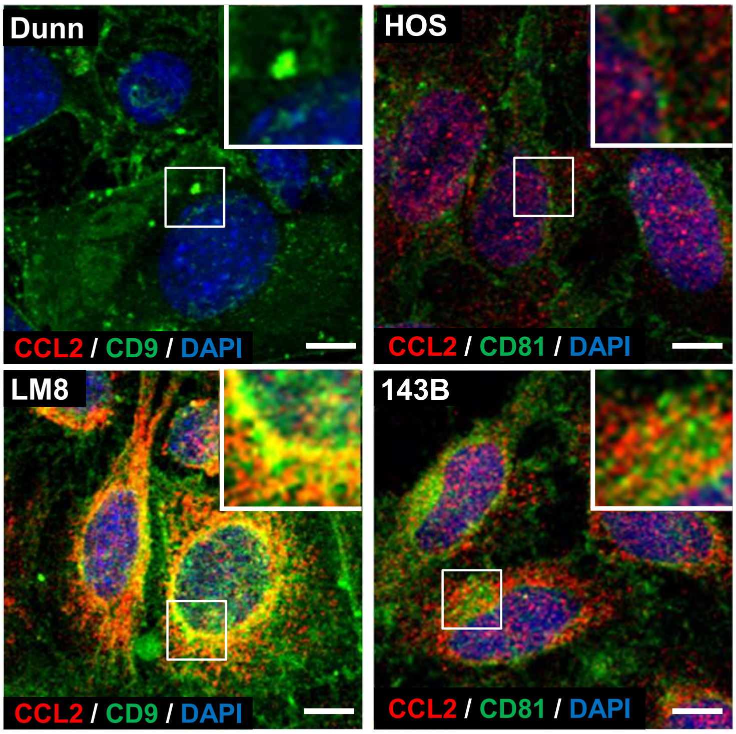

Supplementary Figure S4. Immunocytochemistry analysis of CCL2 and EV surface markers (CD8, CD81) using mouse and human OS cells. Scale bars, 10 µm.

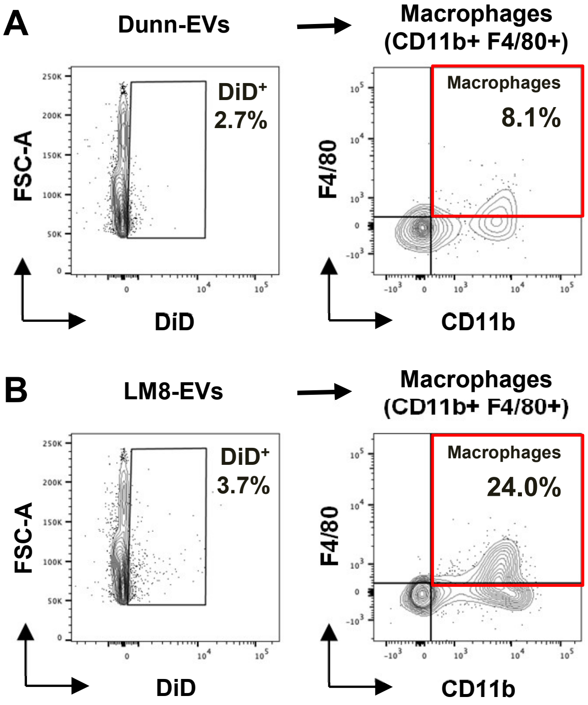

Supplementary Figure S5. Gating strategy for macrophages containing fluorescence-labeled EVs in FACS analysis. A, **B** The extracellular vesicles (EVs) were collected from the conditioned medium of nonmetastatic Dunn cells (**A**) and metastatic LM8 cells (**B**) and labeled using Vybrant DiD Cell-Labeling Solution. The fluorescence-labeled EVs were intravenously injected and tissues were harvested for FACS analysis. Representative FSC, SSC, CD11b, and F4/80 plots with gating for macrophages containing DiD+ EVs.

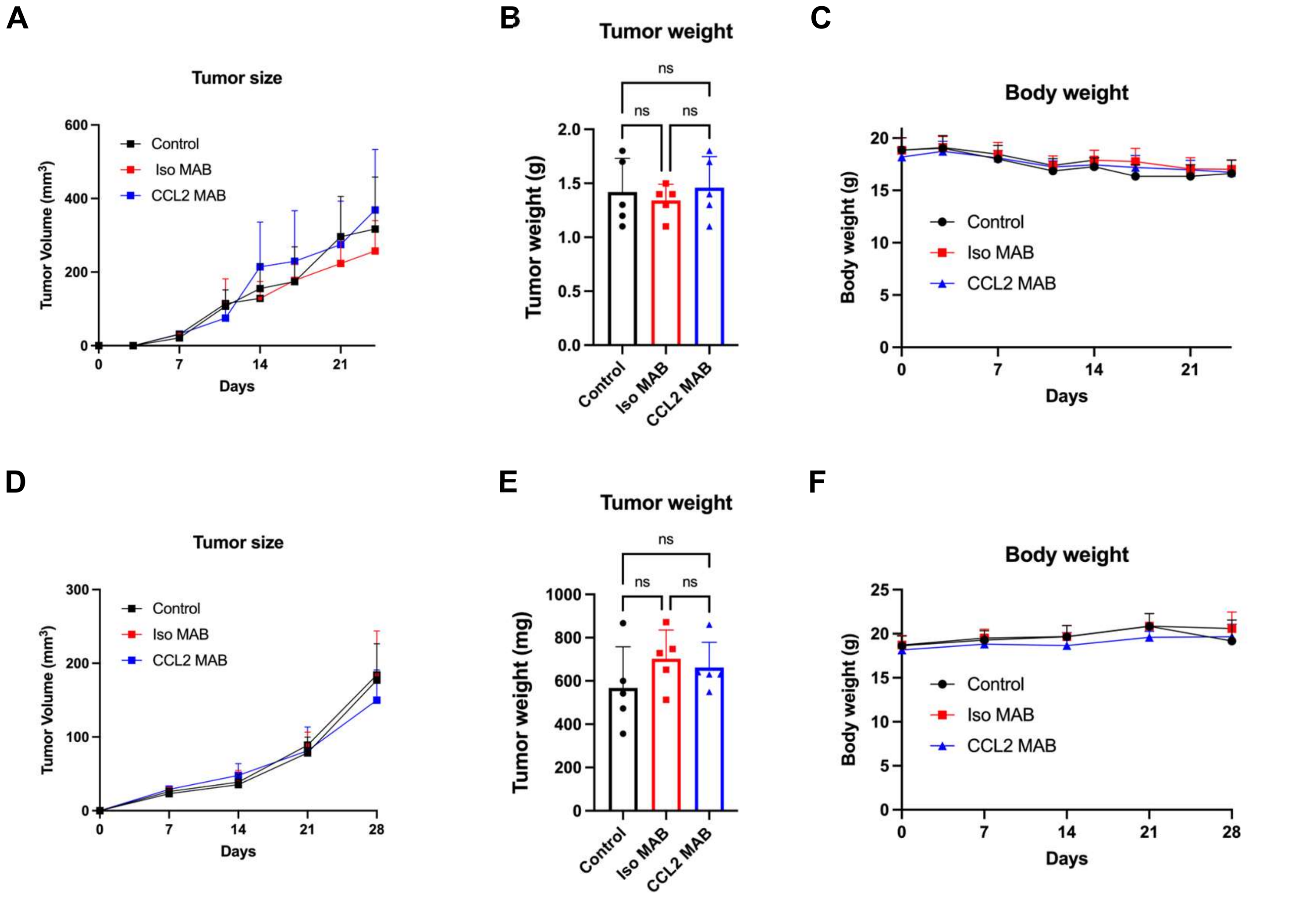

Supplementary Figure S6. Tumor growth curves, tumor weights, and body weights in orthotopic tumor models with metastatic OS cells after treatment with CCL2-neutralizing antibody. Murine LM8 cells or human 143B cells with metastatic abilities (2×10^6 cells) were inoculated orthotopically into the tibia of C3H/He mice or nude mice, respectively. Anti-mouse/human CCL2 antibody (CCL2 MAB) or isotype control IgG (Iso MAB) were injected intraperitoneally into the tumor-bearing mice. **A**, Growth curves of LM8 tumors. **B**, Mean weights of LM8 tumors on day 24. **C**, Mean body weights of LM8 tumor-bearing mice. **D**, Growth curves of 143B tumors. **E**, Mean weights of 143B tumors on day 28. **F**, Mean body weights of 143B tumor-bearing mice. Data are expressed as mean ± SD (n = 5 in each group; ns, not significant).

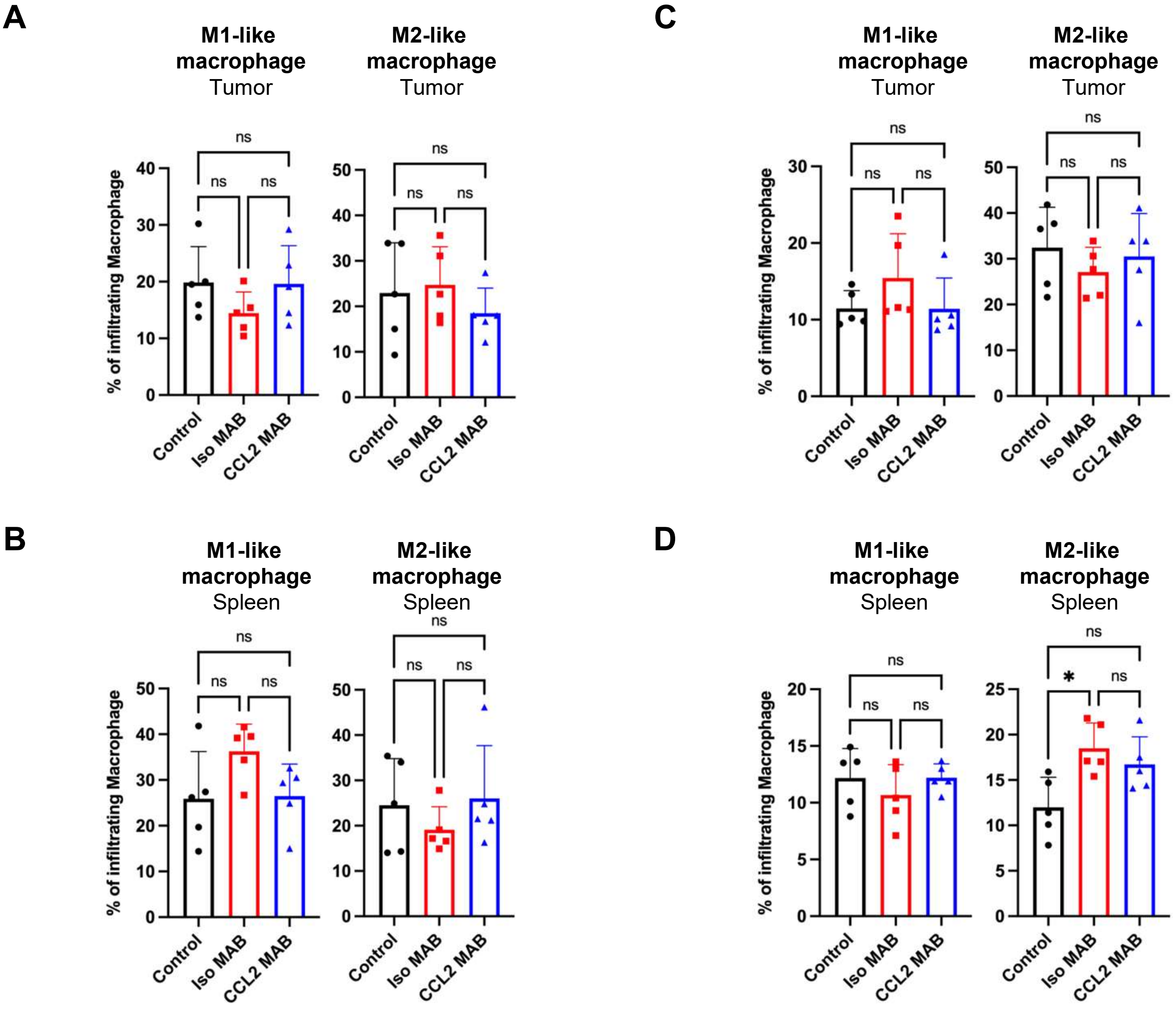

Supplementary Figure S7. Proportion of M1-like and M2-like macrophages in primary tumors and spleens of mice bearing metastatic OS tumors after treatment with CCL2-neutralizing antibody. LM8 cells or human 143B cells with metastatic abilities (2×10^6 cells) were inoculated orthotopically into the tibia of C3H/He mice or nude mice, respectively. Anti-mouse/human CCL2 antibody (CCL2 MAB) or isotype control IgG (Iso MAB) were injected intraperitoneally into the tumor-bearing mice. **A**,**B**, The proportions of M1 and M2 macrophages in primary tumors (A) and spleens (B) of mice orthotopically inoculated with LM8 cells. **C**,**D**, The proportions of M1 and M2 macrophages in primary tumors (C) and spleens (D) of mice orthotopically inoculated with143B cells. Data are expressed as mean ± SD (n = 5 in each group; *, $P < 0.05$; ns, not significant).
